# The JIL-1 Kinase Affects Telomere Expression in the Different Telomere Domains of *Drosophila*

**DOI:** 10.1371/journal.pone.0081543

**Published:** 2013-11-14

**Authors:** Rute Silva-Sousa, Elena Casacuberta

**Affiliations:** Institute of Evolutionary Biology, IBE (CSIC-UPF), Barcelona, Spain; University College London, United Kingdom

## Abstract

In *Drosophila*, the non-LTR retrotransposons *HeT-A*, *TART* and *TAHRE* build a head-to-tail array of repetitions that constitute the telomere domain by targeted transposition at the end of the chromosome whenever needed. As a consequence, *Drosophila* telomeres have the peculiarity to harbor the genes in charge of telomere elongation. Understanding telomere expression is important in *Drosophila* since telomere homeostasis depends in part on the expression of this genomic compartment. We have recently shown that the essential kinase JIL-1 is the first positive regulator of the telomere retrotransposons. JIL-1 mediates chromatin changes at the promoter of the *HeT-A* retrotransposon that are necessary to obtain wild type levels of expression of these telomere transposons. With the present study, we show how JIL-1 is also needed for the expression of a reporter gene embedded in the telomere domain. Our analysis, using different reporter lines from the telomere and subtelomere domains of different chromosomes, indicates that JIL-1 likely acts protecting the telomere domain from the spreading of repressive chromatin from the adjacent subtelomere domain. Moreover, the analysis of the 4R telomere suggests a slightly different chromatin structure at this telomere. In summary, our results strongly suggest that the action of JIL-1 depends on which telomere domain, which chromosome and which promoter is embedded in the telomere chromatin.

## Introduction

*Drosophila* telomeres are the best-studied telomerase exception to date. The transposition of three special non-LTR retrotransposons; *HeT-A*, *TART* and *TAHRE* (HTT), buffers the receding chromosome ends when needed [1,2]. Therefore, in *Drosophila* the genes responsible for telomere elongation are embedded in the telomere chromatin and to understand telomere regulation in this organism it is necessary to understand gene expression from this genomic compartment.

*Drosophila* telomeres are organized in two different domains; the *cap* domain protecting the very end of the chromosome, and the *telomere* domain composed of the HTT array. Additionally, immediately adjacent to the HTT array exists the *subtelomere* domain, composed of the Telomere Associated Sequences (TAS) [3-5]. Although the subtelomere domain is not strictly part of the telomere, its closeness to the HTT array influences some of its functions.

Previous studies, based on the variegation of reporter genes, suggested that TAS sequences nucleate a compacted chromatin restrictive of gene expression at the telomeres, causing a telomere position effect (TPE) [6]. Later, molecular studies detected the presence of chromatin marks typical of repressive and compacted chromatin in TAS, such as H3K27me3 and Polycomb [3,4,6,7]. 

We aimed to better understand the regulation of gene expression in *Drosophila* telomeres. With this objective we assayed the influence of the JIL-1 kinase over *Drosophila* telomere and subtelomere domains. In *Drosophila*, JIL-1 is an essential gene responsible for the phosphorylation of the Ser10 of Histone H3 when cells are in interphase or when a stress response is needed [8-10]. Besides the telomeres, JIL-1 localizes at the interband region of polytene chromosomes and has been shown to be involved in maintaining chromosome structure and in protecting genomic regions from an excess of heterochromatinization [11-15]. JIL-1 has been found to localize among other genomic locations, at the HTT array and to be the first positive regulator of the expression of the telomeric retrotransposons [3,16]. In this study, we report that JIL-1 protects the telomere domain from the spreading of repressive chromatin from the flanking subtelomere domain and that JIL-1 action depends on which telomere domain, which chromosome and which promoter is embedded in the telomere chromatin. 

## Results

In order to assess the possible role of JIL-1 regulating gene expression at the HTT array, we chose several lines that contain a mini-white reporter gene at the telomere domain (HTT array), or at the subtelomere domain (TAS sequences) from different chromosomes [6,17,18]. The mini-white gene from the chosen reporter lines is expressed from the same basic promoter, which does not include the eye-specific enhancers (*see references above*). All flies in this study are in a *white* minus background (*w*-, white eyes). The amount of red color that is observed in the eyes of the reporter lines (EY08176, EY00453, EY09966, 39C-27, 39C-5, 39C-72 and 118E-5), depends on where in the chromosome the mini-white gene has been inserted. In order to evaluate the effect of the different mutations assayed in this study over the reporter lines, we have compared the descendants of the crosses with the *JIL-1* and the *Su*(*var*)*2-5* mutant flies (also in a *w*- background and with white eyes) with the descendants resulting from crossing the flies containing the reporter lines with a *w* ^1118^ strain (white eyes). This control cross is necessary in order to be able to evaluate the presence of the reporter gene in heterozygous state. We search for changes in eye color revealing a TPE effect.

### JIL-1 is necessary for gene expression at the telomere domain (HTT)

**Figure 1:**
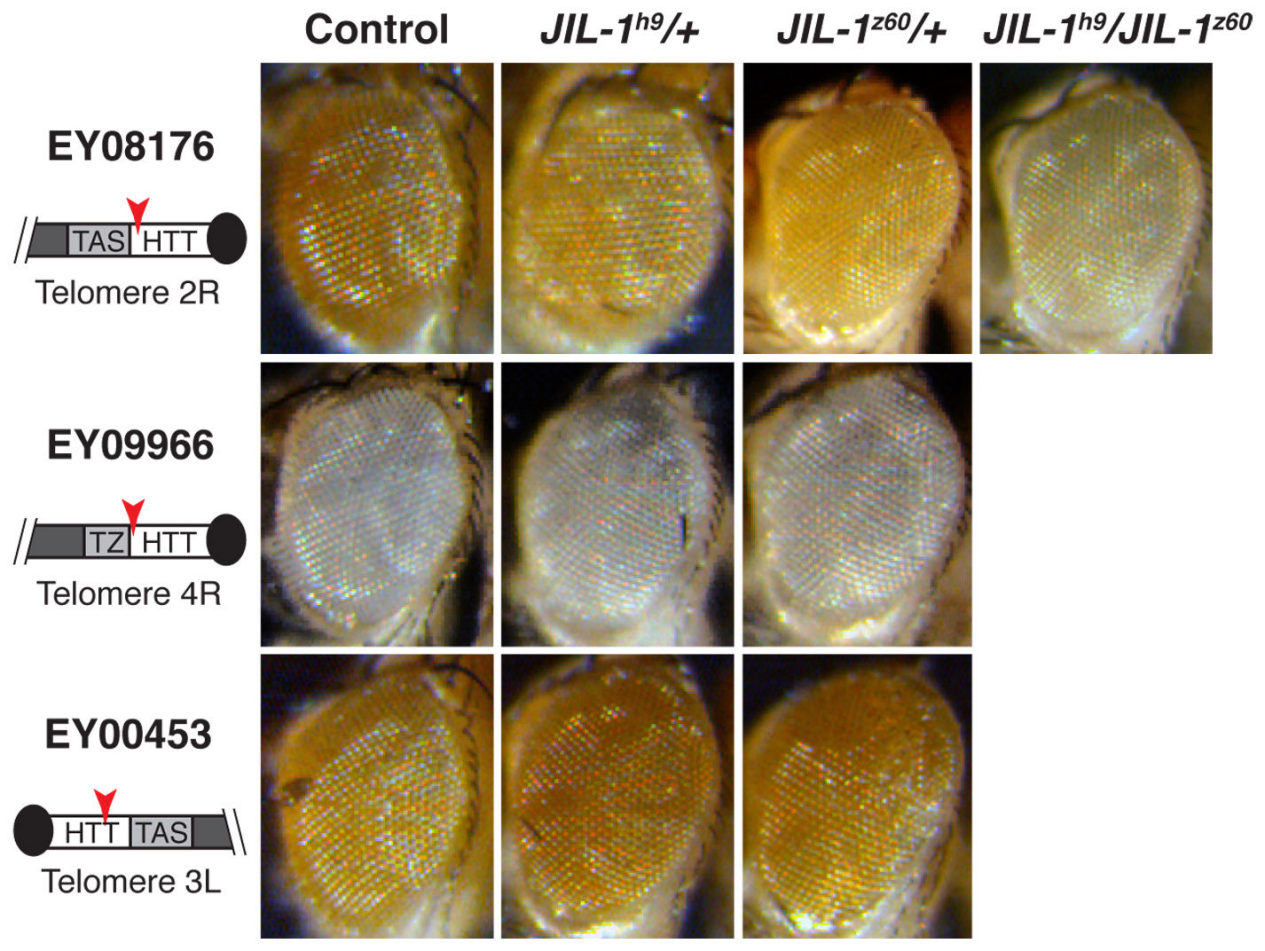
JIL-1 affects the expression of genes embedded in the HTT array. For all crosses, eyes from F1 males are shown. 1^st^ column: control: F1 male descendants from crossing *w* ^1118^ females with male flies with the indicated reporter line, 2^nd^ and 3^rd^ columns: results from the crosses of the *JIL-1* heterozygous, *JIL-1* ^*z60*^/+ or *JIL-1* ^*h9*^ */+*, with the indicated reporter line. 4^th^ column: Result from crossing the *JIL-1^z60^/JIL-1* ^*h9*^ transheterozygous allele with the indicated *mini-white* insertion. The schematic drawings on the left indicate the position of the insertion (red arrow head) of the mini-white reporter gene in each of the reporter lines. Black circle represents telomere cap, white rectangle the telomeric retrotransposon array (HTT), light grey rectangle the subtelomeric domain TAS, telomere associated sequences, or TZ, transition zone, and dark grey rectangle represents the chromosome arm. EY08176 for the 2R telomere (1^st^ row), EY09966 for the 4R telomere (2^nd^ row), and EY00453 for the 3L telomere (3^rd^ row). Note that descendants from the *JIL-1* heterozygous, *JIL-1* ^*z60*^/+ or *JIL-1* ^*h9*^ */+*, show a subtle enhancer effect in the telomere line EY08176 (1^st^ row) and no effect with the telomere line EY00453 (3^rd^ row). We did not observe any effect in the EY09966 line corresponding to the 4R telomere, likely because the reporter gene is already heavily repressed in this line (2^nd^ row). When the *JIL-1^z60^/JIL-1* ^*h9*^ transheterozygous allele is assayed with the EY08176 reporter line, an enhancer effect of TPE is observed (4^th^ column).

By crossing flies containing mutant alleles of *JIL-1* with lines with a mini-white gene inserted at the HTT array from different chromosomes, we screened for TPE effects in the eyes of the first generation (Figure 1). In simple heterozygous combinations of *JIL-1* alleles (Figure 1, 2^nd^ and 3^rd^ columns), we observed subtle enhancer position effect variegation in the telomere line EY08176 (1^st^ row) and no effect with the telomere line EY00453 (3^rd^ row). We did not observe any effect in the EY09966 line corresponding to the 4R telomere, likely because the reporter gene is already heavily repressed in this line (2^nd^ row). Nevertheless, when we combined both *JIL-1* alleles in a trans-heterozygous mutant (*JIL-1z60*/*JIL-1* ^*h9*^) with the line EY08176, a clear enhancer effect of TPE was revealed (1^st^ row, 4^th^ column). 

**Figure 2:**
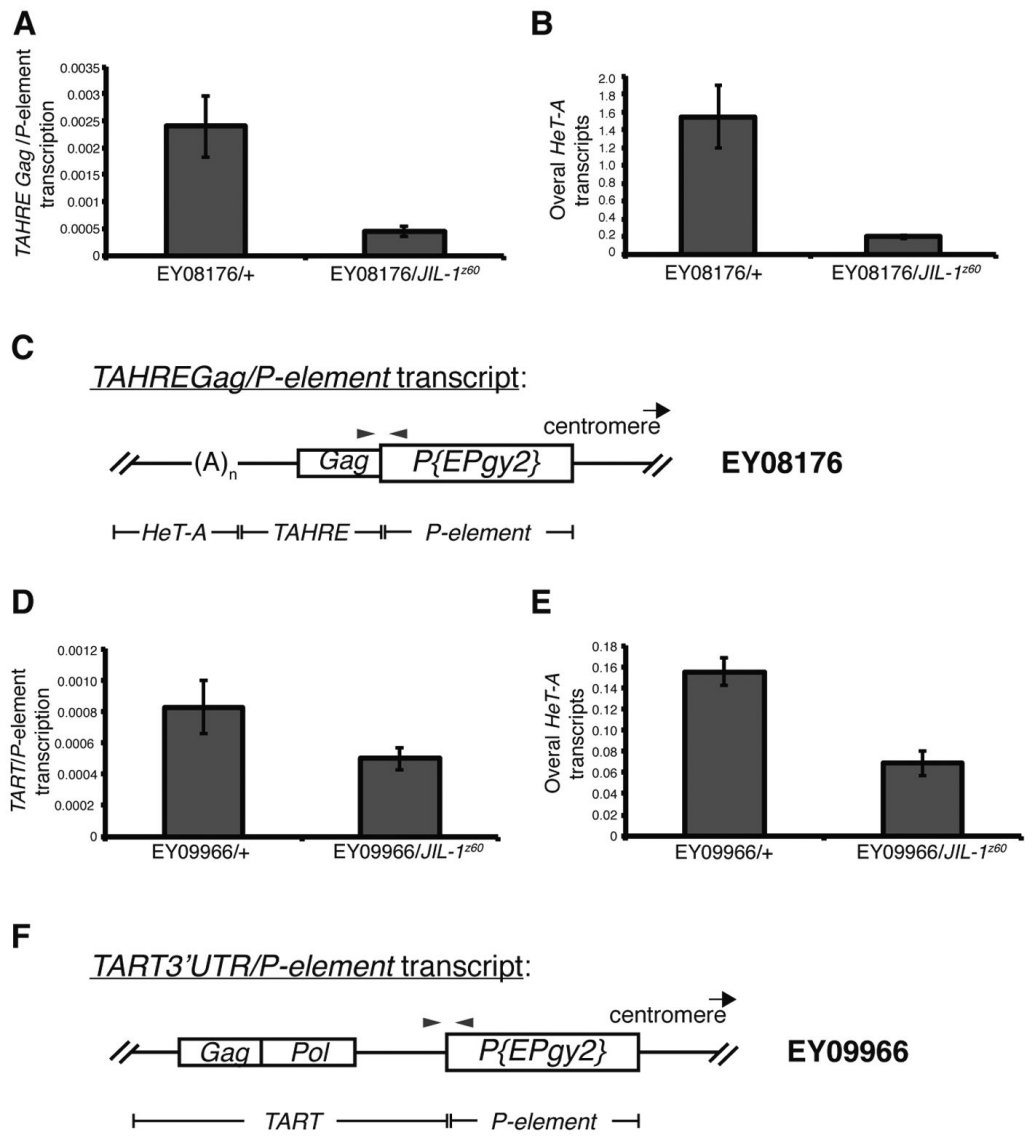
Quantification of the decrease in telomere expression in *JIL-1* mutants. *JIL-1* mutations lead to a decrease in the transcription of the telomeric retrotransposon immediately downstream of the *P*-element insertion (**A** and **D**), as well as in the overall *HeT-A* transcripts (**B** and **E**). Primers used to quantify overall *HeT-A* transcripts anneal at the 3’UTR region of the element (see M&M for more details). (**C** and **F**) correspond to a schematic representation of the telomeric retrotransposon/*P-element* transcript analyzed by quantitative real-time PCR in A and D, respectively. Primers shown with grey arrowheads indicate the fragment amplified and quantified.

Following, we investigated if, in comparison with the expression in the wild type situation of the reporter line, a decrease in the expression of the HTT retroelements upstream to the mini-white insertion was observed in *JIL-1* mutants. Quantitative real-time PCR was used to detect expression of the transcript containing both, the telomeric element and the mini-white reporter (Figure 2A and D). When compared with the expression detected in the reporter in a wild type background, *JIL-1* heterozygous mutants show lower telomere transcription from the HTT element adjacent to the insertion in the 2^nd^ and in the 4^th^ chromosomes (EY08176, Figure 2A and D) (EY09966, Figure 2B and F). This reduction is in agreement with a reduction of the overall expression of *HeT-A* in the same reporter lines (Figure 2B, 2D and [16]) suggesting a global role of JIL-1 over the HTT array. 

**Figure 3:**
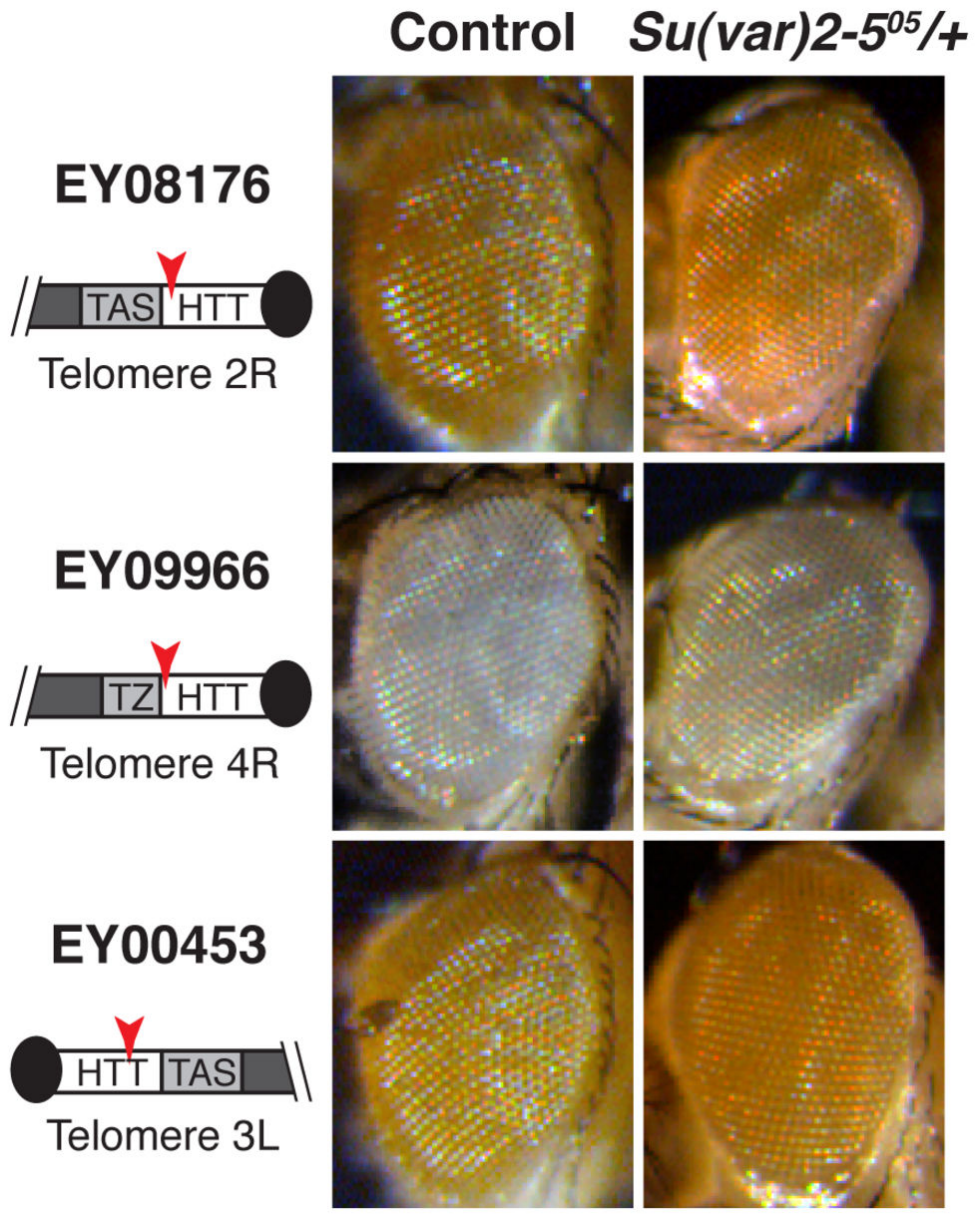
Effect of HP1 over genes embedded at the HTT array. *Su*(var)*2-5* ^*05*^, a HP1a mutant allele, was assayed with the same reporter lines from Figure 1. Although it has been demonstrated that *Su*(var)*2-5* ^*05*^ shows a strong de-repression of *HeT-A* transcription (see main text), no substantial change in the expression of the mini-white reporter is detected in the telomeric lines (1^st^, 2^nd^ and 3^rd^ rows). As in Figure 1, schematic drawings on the left. Black circle represents telomere cap, white rectangle the telomeric retrotransposon array (HTT), light grey rectangle the subtelomeric domain TAS, telomere associated sequences, or TZ, transition zone, and dark grey rectangle represents the chromosome arm.

In addition, we assayed the possible effect of flies containing mutant alleles of the *Su*(*var*)*2-5* gene (HP1a), over the reporter lines inserted at the HTT array (Figure 3). Interestingly, only a subtle change in mini-white expression was detected when we assayed the *Su*(*var*)*2-5* ^05^ mutation over the lines EY08176, EY09966 and EY00453 (Figure 3 and [18]). 

### JIL-1 acts as a suppressor of TPE over the TAS domain

**Figure 4:**
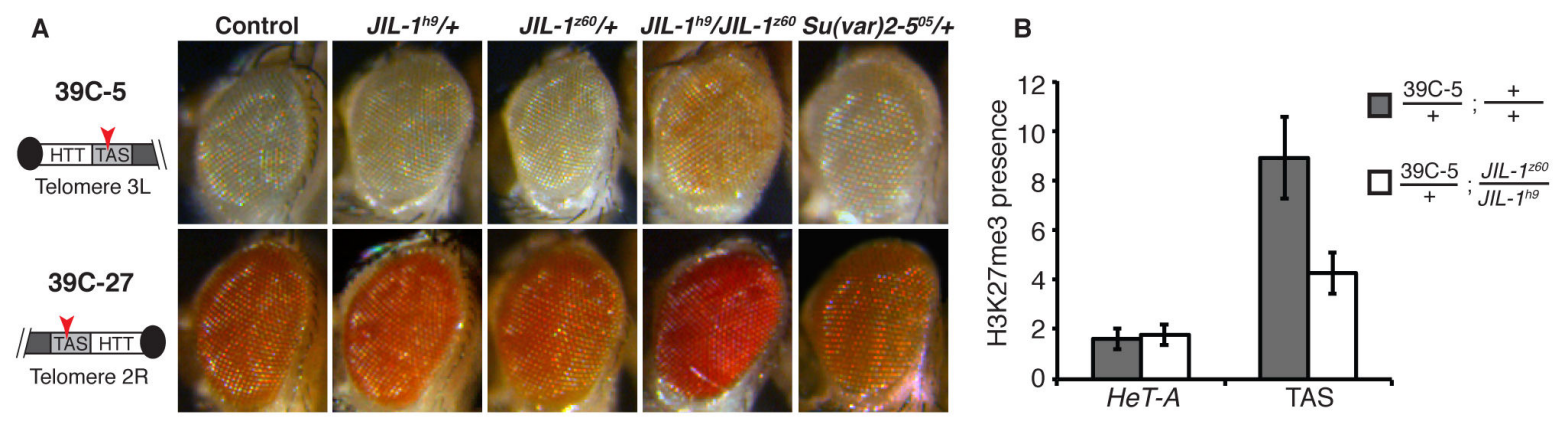
JIL-1 act as suppressor of TPE over the TAS domain. (**A**) TPE assays in the TAS domain were performed with the reporter lines 39C-5 (chromosome 2L, TAS-L) and 39C-27 (chromosome 2R, TAS-R). No effect on the mini-white gene expression was observed when the reporter lines were crossed with the *JIL-1* heterozygous mutants *JIL-1* ^*z60*^/+ and *JIL-1* ^*h9*^ */+* (2^nd^ and 3^rd^ column). However, when the same lines were crossed with the trans-heterozygous mutant allele (*JIL-1z60*/*JIL-1* ^*h9*^), a clear increase in the eye color was observed revealing a suppressor effect of JIL-1 over the TAS domain (4^th^ column). 5^th^ column: control cross of the TAS reporter lines with the *Su*(var)*2-5* ^*05*^, a HP1a mutant allele, does not show any differences in the expression of the mini-white gene inserted in TAS as expected by the literature (see main text). (**B**) Chromatin immunoprecipitation (ChIP) experiments reveal that the *JIL-1* trans-heterozygous mutant allele (*JIL-1z60*/*JIL-1* ^*h9)*^ lead to a decrease in the presence of the H3K27me3 mark at the TAS domain. Three independent ChIP samples were analyzed and the amount of immunoprecipitated DNA was calculated by three independent quantitative real-time PCRs. Specific primers were used to quantify the amount of TAS or *HeT-A* DNA immunoprecipitated by the H3K27me3 antibody (see *M*&*M*).

*JIL-1* ^*z60*^/+ and *JIL-1* ^*h9*^/+ heterozygous alleles did not produce an appreciable effect over the mini-white inserted at the TAS domain of chromosomes 2R (39C-27) and 2L (39C-5) (Figure 4A, 2^nd^ and 3^rd^ columns). Nevertheless, when the dose of JIL-1 was lowered in a trans-heterozygous combination, a significant increase in the mini-white expression was observed when compared with the control cross (Figure 4A 1^st^ column), revealing a suppressor effect of JIL-1 over the TAS domain of these two telomeres (Figure 4A, 4^th^ column). As expected, we did not observe any change in mini-white expression when the TAS lines (39C-27 and 39C-5) were combined with the HP1a allele *Su*(*var*)*2-5* ^*05*^ (Figure 4A, 5^th^ column) [7].

**Figure 5:**
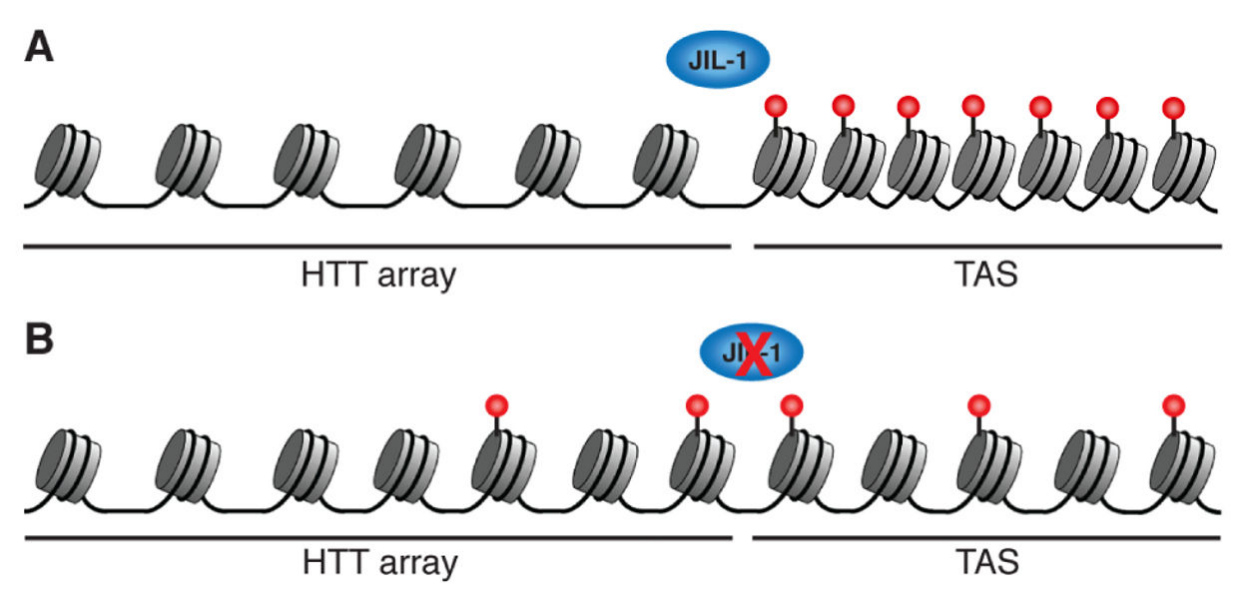
Possible scenarios for the TAS and the HTT chromatin in a *JIL-1* mutant background. (**A**) Wild type: the presence of the H3K27me3 mark nucleates a highly repressed chromatin (note the tight packaging of the nucleosomes) in the TAS domain, regulated by the Polycomb group of proteins (Table S1). (**B**) *JIL-1* mutant background: The loss of the JIL-1 boundary, causes a spread of the H3K27me3 mark into the HTT array, resulting in a local decrease of the H3K27me3 mark in the TAS chromatin and as a consequence making this compartment more permissive to gene expression.

In order to understand which changes in the TAS chromatin could explain the increase in mini-white transcription in the *JIL-1^z60^/JIL-1* ^*h9*^ allelic combination, we performed Chromatin Immunoprecipitation (ChIP) experiments. The chromatin environment at the TAS domain has been shown to be enriched in H3K27me3, which serves for targeting the Polycomb complex to the subtelomere domain [3,7]. We therefore developed the ChIP assay with an antibody against H3K27me3 (Figure 4B). The ChIP data showed a significant decrease in the presence of the H3K27me3 mark inside the TAS domain in *JIL-1* mutant alleles, suggesting a possible spreading of this mark into flanking domains. We have not been able to detect an increase in H3K27me3 at the HTT array indicative of the consequential spreading of this mark from the TAS towards the HTT array, (Figure 4B and 5). Because the HTT array flanking the TAS domain at the 2R (39C-27) and 2L (39C-5) telomeres cannot be distinguished from the HTT arrays of other chromosomes, it is not surprising that the ChIP assay was not sensitive enough to reveal a slight increase in H3K27me3 in the vicinity of the HTT array. 

The TPE assays with the *JIL-1* mutant alleles have shown the same result for both TAS domains, TAS-L (2L, 3L telomeres, line 39C-5) and TAS-R (2R, 3R and XL telomeres, line 39C-27) [19], demonstrated by the increase in eye color of the resulting descendants (Figure 4A). These results suggest that the protective boundary built by JIL-1 also exists in the other *Drosophila* telomeres. 

As an additional control, we analyzed if the TAS reporter lines 39C-27 and 39C-5 behave as had been previously described for TAS sequences [6,7]. Therefore, we performed control crosses with mutant alleles of genes whose effect over the expression in the subtelomeric domain had been already reported (Table S1) and obtained results consistent with previous studies [6,7]. 

### The subtelomere domain of the 4^th^ chromosome and the TAS domains have a different chromatin

**Figure 6:**
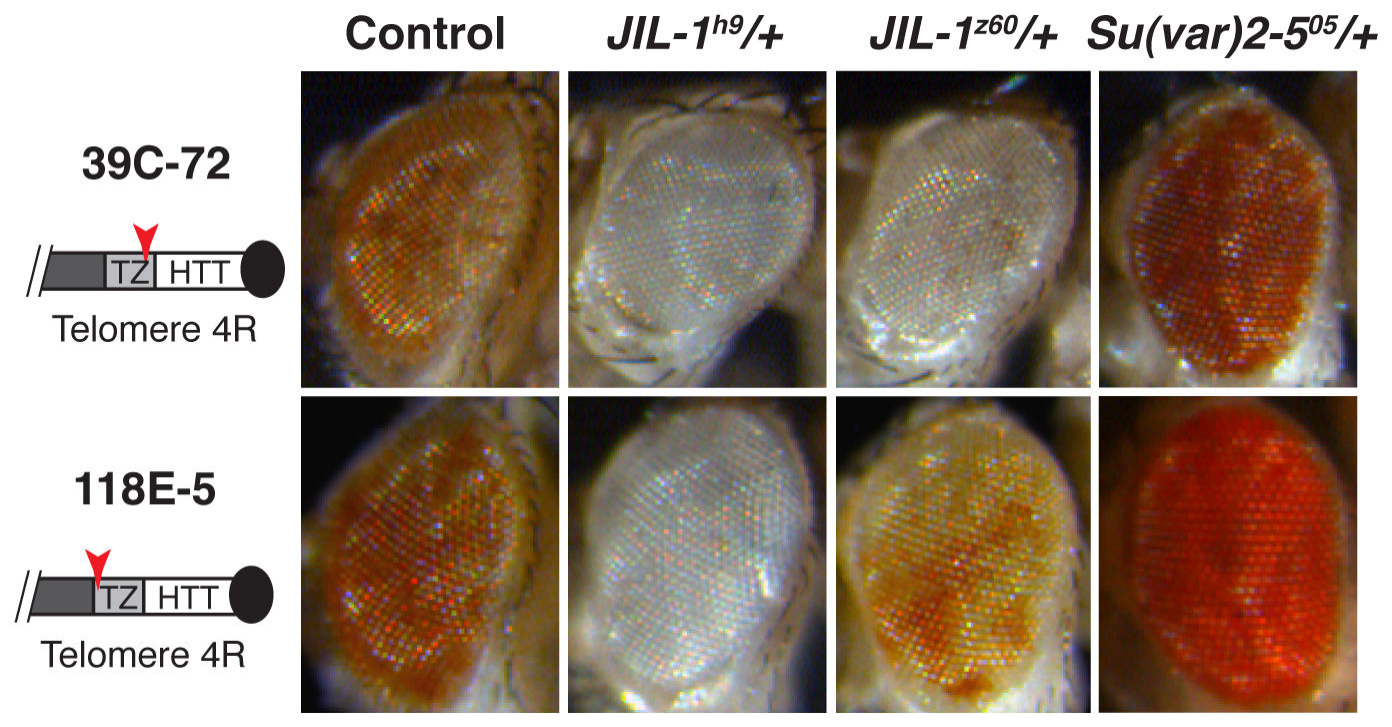
JIL-1 and HP1 control expression from the subtelomeric region of the fourth chromosome. Reporter lines from the subtelomere domain (transition zone) of chromosome 4R, 39C-72 and 118E-5, were crossed with the heterozygous mutant alleles *JIL-1* ^*z60*^/+, *JIL-1* ^*h9*^ */+* and *Su*(var)*2-5* ^*05*^. The subtelomere domain of the 4^th^ chromosome shows a clear descent in eye color already with the simple *JIL-1* heterozygous alleles, (2^nd^ and 3^rd^ columns) and a strong suppressor effect of TPE in the *Su*(var)*2-5* ^*05*^ allele (4^th^ column).

The 4R telomeres of the reporter lines here analyzed (39C-72 and 118E-5) do not have a TAS domain flanking the HTT array. Instead, a small transition zone (≈ 5Kb) composed of decayed transposable elements together with pieces of the HTT elements lies at the subtelomere region of the 4R arm (*Flybase GBrowse* and [6,20]). Our experiments have revealed that the JIL-1 kinase and HP1a easily affect the 4^th^ chromosome subtelomeric lines 39C-72 and 118E-5 (Figure 6). The effects that these two proteins exert at the 4R subtelomere are similar to the ones that they produce at the HTT array of the other chromosomes (Figure 1 4^th^ column and Figure 2A, B, D, E and Figure 3). JIL-1 behaves as an enhancer of TPE while HP1a shows a strong suppressor effect in all cases where the reporter is inserted at the telomere domain. Additionally, we performed control crosses with insertions inside the arms of the 4^th^ chromosome, the lines 39C-12 and 39C-42 [21]. No change in the eye color of the *JIL-1* mutant descendants was observed, while in *Su*(*var*)*2-5* ^*05*^ mutants the expected increase in red eye color with respect to the control cross was observed (Figure S1) [21]. Therefore, our results suggest that the subtelomere of the 4^th^ chromosome depends on JIL-1 for gene expression and on HP1a for gene silencing (Figure 6).

## Discussion

### JIL-1 is a positive regulator of gene expression at the telomere domain (HTT)

Both the experiments of telomere position effect on the mini-white insertions (Figure 1) and the real-time PCR quantifications of the expression of the different telomeric elements (Figure 2), indicate that the lower amount of JIL-1 present in the mutant alleles *JIL-1* ^*z60*^ and *JIL-1* ^*h9*^ here assayed, results in a decreased expression of the HTT array. These experiments, in accordance with our recent published data [16], confirm that JIL-1 is necessary to obtain wild type levels of gene expression from the HTT array. 

Additionally, these experiments have also revealed that the effect of JIL-1 is stronger over the *HeT-A* promoter than over the mini-white promoter existent in the reporter lines used in this study. Similarly, when we assayed the effect of mutations of the *Su*(*var*)*2-5* gene, known to greatly de-repress the expression of the *HeT-A* retrotransposon [16,22-24], we only obtained a faint de-repression of the mini-white gene of the lines EY08176, EY09966 and EY00453 (Figure 3). In agreement with our observations, Frydrychova and colleagues found similar results using two additional *Su*(*var*)*2-5* mutant alleles, *Su*(*var*)*2-5* ^02^ and *Su*(*var*)*2-5* ^*04*^, over the same reporter lines [23]. 

In summary, JIL-1 and HP1a control gene expression from the telomere domain in *Drosophila*, being the *HeT-A* promoter especially sensitive to their effect. 

### JIL-1 protects the HTT array of the chromatin spreading from the TAS domain

Although JIL-1 has not been found to localize at the subtelomere domain, TAS, the observation that gene expression varies in this domain in a *JIL-1* mutant background, suggests a role of this protein related with a putative boundary between the telomere and subtelomere domains. We have observed a pronounced increase in mini-white expression at the TAS domains from the 2L and 2R telomeres when placed in a *JIL-1* trans-heterozygous (*JIL-1^z60^/JIL-1* ^*h9*^) mutant background (Figure 4A, 4^th^ column). Accordingly, the ChIP data reveals a significant decrease of H3K27me3 upstream of the mini-white insertion when a *JIL-1* trans-heterozygous background is present (Figure 4B). Integrating these results, we propose a model in which the JIL-1 kinase acts as a boundary protecting the promoters of the HTT retrotransposons from the highly compacted chromatin of the adjacent TAS domain (see model in Figure 5). This model is in agreement with an increase of repressive chromatin at the HTT array in *JIL-1* mutations (Figure 1 4^th^ column and [16]). Moreover, our model also reflects the previously reported role of JIL-1 in the protection of the excessive spreading of heterochromatin to adjacent domains [13,14], and suggests that JIL-1 could exert a barrier function at the HTT-TAS boundary, based on the results obtained from this study.

### Not all the subtelomere domains have the same chromatin characteristics

We have not observed a different behavior in the control of gene expression when studying the HTT array of the 4^th^ and the 2^nd^ chromosomes. Interestingly, we did find a significant difference when looking at the subtelomere of the 4R telomere (Figure 6). In the reporter lines 39C-72 and 118E-5 from the subtelomere domain of the 4^th^ chromosome, we have found that JIL-1 is important to allow gene expression and HP1a is necessary to repress it. This finding indicates that the chromatin of the subtelomere domain in the 4^th^ chromosome is not equivalent to the TAS chromatin in the other chromosomes.

In contrast, the results here described indicate that the chromatin at the 4R subtelomere of the reporter lines used in this study is less compacted and more permissive to gene expression than the TAS domains from the other chromosomes, and even than the HTT array. This scenario suggests that in the 4R telomere of these lines the compaction of the chromatin is in the opposite orientation than in the rest of the telomeres. It would be interesting to study if this reversed chromatin organization with respect to the other telomeres has a particular role in the general telomere function in *Drosophila*. 

## Conclusions

We report how the presence of the JIL-1 kinase is needed at the telomere in *Drosophila* in order to allow gene expression of the promoters embedded in this domain. The lack of JIL-1 causes an increase of silencing at the HTT array and a release of silencing at the subtelomeric domain likely by the spreading of heterochromatin towards adjacent domains. Finally, we discovered that the telomere and subtelomere domain of the 4R arm of some lines might have a different chromatin organization with respect to the other *Drosophila* telomeres. 

## Materials and Methods

### Fly stocks and crosses

Fly stocks were maintained and crosses performed at 25°C on standard *Drosophila* corn meal medium. *JIL-1^z60^/TM6* and *JIL-1^h9^/TM6* stocks were provided by Kristin M. Johansen. Both *JIL-1* mutations are hypomorph. The *JIL-1* ^*h9*^ allele contains a molecular lesion that results in a deletion inside the C-terminal of the protein and the *JIL-1* ^*z60*^ contains a mutation that eliminates the promoter and 5’UTR of the *JIL-1* gene [9]. *Su*(*var*)*2-5* ^*05*^ contains a frame shift that results in a peptide containing only the first 10 amino acids of the original HP1 protein [22]. *w* ^1118^, *Su*(*var*)*2-5^05^/CyO*, *Su*(*Z*)*21*.*a1*/CyO and *Su*(*Z*)*21*.*b7*/CyO were obtained from Bloomington Stock Center. *Su*(*var*)*3-917*/TM3 and *Bmx2trx/TM6* were provided by Fernando Azorin laboratory. Lori Walrath provided the reporter lines 39C-5, 39C-27, 118E-5, 39C-72, 39C-42, and 39C-12, and James Mason the lines EY08176, EY00453 and EY09966. 

To assay for TPE modifications, males from homozygous reporter gene stocks, with a *P*-element inserted at a telomere or at the subtelomere domains, were crossed with virgin females from mutant stocks of the proteins of interest. The eye phenotype of the resulting F1 progeny with the mutation was compared with their siblings lacking the mutation. For each cross 20 flies were analyzed (males and females) and collected 2 days after eclosion and briefly frozen. Each cross was repeated three times. Images were obtained from mutant and control F1 males using Zeiss Discovery V.8 microscope with AxioCam MRE5 camera and Axiovision 4.7 software. 

### RNA Extraction and cDNA synthesis

Total RNA was isolated from ten whole third instar larvae and extracted using RNeasy Mini Kit (Qiagen) according to manufacturer’s protocol. RNase Free DNase Set (Qiagen) was used to remove genomic DNA contaminations as follows: one on column during the extraction accordingly to manufacturer’s protocol, and two in solution for 2 hours at 37°C. RNA was cleaned by precipitation and its quality was assessed using NanoDrop spectrophotometry.

One microgram of RNA was reverse transcribed into cDNA using Transcriptor First Strand cDNA Synthesis Kit (Roche) with Oligo d(T) primers, and the expression of the different transcripts analyzed by quantitative real-time PCR. For each fly strain, two independent RNA extractions were prepared and analyzed three independent times. Primers used for real time PCR: HeT-A_F (CCCCGCCAGAAGGACGGA) and HeT-A_R (TGTTGCAAGTGGCGCGCA) included in the 3’UTR, EY0176_F (GTGGATGTCTCTTGCCGACG) and EY8176_R (GCTCTTACTCACACTGACGCTTCTC), EY09966_F (CTATAACATCGAGTACCAGCCG) and EY09966_R (GCTCTTACTCACACTGACGCTTCTC), Actin_F (GCGCCCTTACTCTTTCACCA) and Actin_R (ATGTCACGGACGATTTCACG).

### Chromatin Immunoprecipitation experiments (ChIPs)

Chromatin immunoprecipitation experiments were preformed has described in [16]. Chromatin was immunoprecipitated with anti-H3K27me3 (ab6002, Abcam). Three independent ChIP samples were analyzed and the amount of immunoprecipitated DNA was calculated by three independent quantitative real-time PCR using iQ^TM^ SYBR^®^ Green Supermix (BioRad). Primers used for quantitative real-time PCR: HeT-A_5’UTR_F (TCGCGCTTCCTCCGGCAT) and HeT-A_5’UTR_R (GCGGTTATTACATTACGGCGG), TAS_X_F (TTGTAATTTGGTGCGGCAGC) and TAS_X_R (CAGCGTGACTGTTCGCATTC), RpL32_F (CAAGAAGTTCCTGGTGCACAA) and RpL32-R (AAACGCGGTTCTGCATGAG).

### Quantitative real-time PCR

Quantitative real-time PCR was performed to determine *HeT-A* expression and in ChIP experiments. The iQ5 Multicolor Real-Time PCR Detection System was used and the iQ^TM^ SYBR^®^ Green Supermix (BioRad) was used to prepare the reactions. Relative levels of *HeT-A* expression were determined using the threshold cycle and normalized to actin levels (or RpL32 for ChIPs). Three independent experiments of two samples each strain were performed.

## Supporting Information

Figure S1 **JIL-1 control crosses.** JIL-1 mutations have no effect on the expression of a reporter gene inserted in the arms of the 4^th^ chromosome (39C-12 and 39C-42). The *Su*(var)2-5^*05*^ allele has the expected suppressor of variegation effect in these same lanes [21].
(TIF)

Table S1 **Subtelomeric domain control crosses.** Previously reported mutant alleles from *su*(*var*)*3-9*, *polycomb* and *trithorax* were crossed with lines containing the reporter gene inserted in the subtelomeric domains of the 4^th^ (39C-72 and 118E-5) and 2^nd^ chromosomes respectively (39C-5 and 39C-27).
(DOC)
