# Supplementary figures and images for: The JIL-1 Kinase Affects Telomere Expression in the Different Telomere Domains of Drosophila
Source: PLoS One. 2013 Nov 14;8(11):e81543. doi: 10.1371/journal.pone.0081543 (PMC3828246; doi:10.1371/journal.pone.0081543)

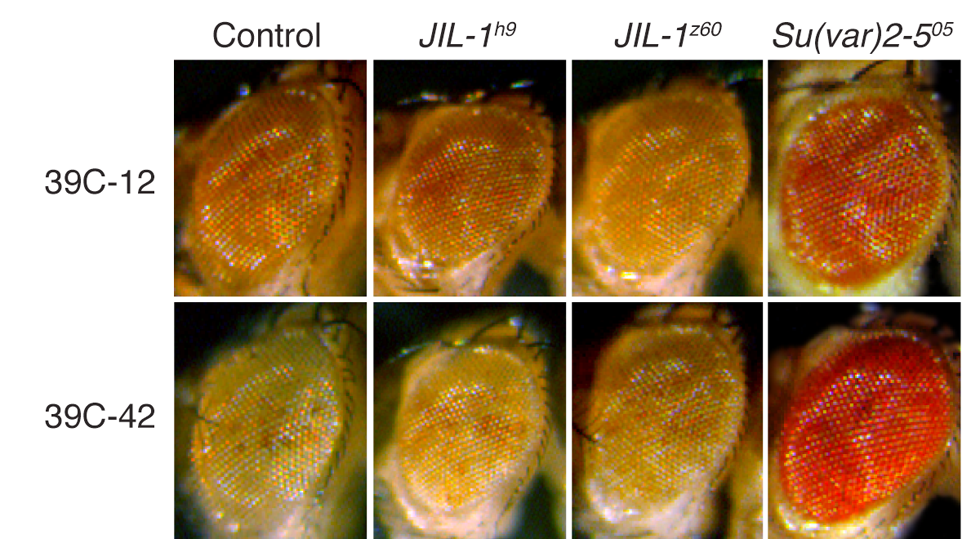

Supplement: Figure S1 — JIL-1 control crosses. JIL-1 mutations have no effect on the expression of a reporter gene inserted in the arms of the 4th chromosome (39C-12 and 39C-42). The Su(var)2-505 allele has the expected suppressor of variegation effect in these same lanes [21]. (TIF) [file pone.0081543.s001.tif]
