# Supplementary material for: The JIL-1 Kinase Affects Telomere Expression in the Different Telomere Domains of Drosophila
Source: PLoS One. 2013 Nov 14;8(11):e81543. doi: 10.1371/journal.pone.0081543 (PMC3828246; doi:10.1371/journal.pone.0081543)
Supplement: Table S1 — Subtelomeric domain control crosses. Previously reported mutant alleles from su(var)3-9, polycomb and trithorax were crossed with lines containing the reporter gene inserted in the subtelomeric domains of the 4th (39C-72 and 118E-5) and 2nd chromosomes respectively (39C-5 and 39C-27). (DOC) [file pone.0081543.s002.doc]

**Supplemental Table 1: Control Crosses**

| **Mutation** | **Effect HTT array** | **Effect TAS** |
| --- | --- | --- |
| *Su(var)3-9^17^* ^(a)^ | No effect | No effect |
| *Su(var)3-9^06e^* ^(a)^ | No effect | No effect |
| *Su(Z)2^1a1^* ^(b)^ | No effect | Suppressor PEV |
| *Su(Z)2^1b7^* ^(b)^ | No effect | Suppressor PEV |
| *Brm^2^trx^E2^ ca^1^* ^(c)^ | No effect | Enhancer PEV |

^(a)^*Su(var)3-9* mutant, ^(b)^*Polycomb* mutant; ^(c)^*Tritorax* mutant.

Lines obtained from the Bloomington stock center: *Brm^2^trx^E2^ ca^1^*, *Su(Z)2^1a1^*, *Su(Z)2^1b7^*. Lines obtained from the laboratory of Fernando Azorín Marin: *Su(var)3-9^17^* and *Su(var)3-9^06e^*.
